# Thermal aerial culling for the control of vertebrate pest populations

**DOI:** 10.1038/s41598-023-37210-0

**Published:** 2023-06-21

**Authors:** Tarnya E. Cox, David Paine, Emma O’Dwyer-Hall, Robert Matthews, Tony Blumson, Brenton Florance, Kate Fielder, Myall Tarran, Matt Korcz, Annelise Wiebkin, Peter W. Hamnett, Corey J. A. Bradshaw, Brad Page

**Affiliations:** 1 Vertebrate Pest Research Unit, New South Wales Department of Primary Industries, 1447 Forest Road, Orange, NSW 2880 Australia; 2 School of Environmental and Rural Science, University of New England, Armidale, NSW 2350 Australia; 3 Aerial Thermal Hunting Services, Murphy Road RD 2, Whakatane, 3192 New Zealand; 4 Heli Surveys, Jindabyne Airport, 56 Tinworth Drive, Jindabyne, NSW 2627 Australia; 5 The Kangaroo Island Landscape Board, 35 Dauncey Street, Kingscote, SA 5223 Australia; 6 Invasive Species Unit, Biosecurity, The Department of Primary Industries and Regions (PIRSA), CSIRO Building 1, Entry 4 Waite Road, Urrbrae, SA 5064 Australia; 7 Global Ecology | Partuyarta Ngadluku Wardli Kuu, College of Science and Engineering, Flinders University, GPO Box 2100, Adelaide, SA 5001 Australia

**Keywords:** Invasive species, Behavioural ecology

## Abstract

Helicopter-based shooting is an effective management tool for large vertebrate pest animals. However, animals in low-density populations and/or dense habitat can be difficult to locate visually. Thermal-imaging technology can increase detections in these conditions. We used thermal-imaging equipment with a specific helicopter crew configuration to assist in aerial culling for feral pigs (*Sus scrofa*) and fallow deer (*Dama dama*) in South Australia in 2021. Seventy-two percent of pigs and 53% of deer were first detected in dense canopy/tall forest habitat. Median time from the first impact shot to incapacitation was < 12 s. The culling rate (animals hour^−1^) doubled compared to visual shoots over the same populations and the wounding rate was zero resulting in a incapacitation efficiency of 100%. The crew configuration gave the shooter a wide field of view and the thermal operator behind the shooter provided essential support to find new and escaping animals, and to confirm species identification and successful removal. The crew configuration allowed for successful target acquisition and tracking, with reduced target escape. The approach can increase the efficiency of aerial culling, has the potential to increase the success of programs where eradication is a viable option, and can improve animal welfare outcomes by reducing wounding rates and the escape of target animals.

## Introduction

Helicopter-based shooting (hereafter ‘aerial culling’) of vertebrate pest animals has been an effective management tool since the 1960s. It has been used to reduce densities of invasive ungulates, most notably in New Zealand where the technique was pioneered to manage introduced deer (red deer *Cervus elaphus scoticus*, wapiti *C. e. nelsoni*, white-tailed deer *Odocoileus virginianus*, sambar deer *C. unicolor*, sika deer *C. nippon*, rusa deer *C. timorensis*, and fallow deer *Dama dama*), goats (*Capra hircus*), and pigs (*Sus scrofa*)^1–3^. In Australia, aerial culling is widely used to control pigs, goats, and increasingly, introduced deer such as fallow, red, sambar, rusa, and chital (*Axis axis*) deer^4,5^. Aerial culling for pigs forms part of routine management operations in the USA^6,7^ and is considered a humane control method for pigs^8,9^ and deer^5,10^. The two primary determinants of good welfare being reduced duration and intensity of animal suffering^11^.

Aerial culling can remove many animals quickly (i.e., days/weeks)^12,13^, can be more effective in remote locations than ground-based shooting^14^, and can be an effective landscape-scale control tool for medium-to-large bodied species^2,14–16^. However, effectiveness depends on visual detection of the target species, which can be difficult in tall forests and/or dense vegetation. The success of aerial culling can also be reduced when the target population size is small^17^ (e.g., an emerging population), or when populations have already been reduced substantially by previous intervention. In artificially reduced populations, survivors of previous aerial culling often flee to the safety of vegetation cover, reducing the efficacy of subsequent control^18,19^.

In New Zealand, aerial culling had the greatest effects on red deer abundances in open habitats such as grasslands and subalpine shrublands^20,21^. However, the aerial control of deer in tall forests has been less successful^20,22^. In Australia, aerial culling was used to remove the last remaining goats from Kangaroo Island^23^ and has been an effective control method for goats in arid environments^24^. However, in forested and rugged terrain, aerial culling of goats was ineffective, with only 31% of known animals culled^19^. Similarly, aerial culling for pigs can reduce populations rapidly and extensively where there is good visibility^16,25–27^. Yet, pigs frequently take cover in thick vegetation, limiting the effectiveness of aerial culling^18^. Low probability of visual detection during aerial culling can result in the mistaken assumption that populations have been reduced to acceptably low numbers, premature cessation of control, and higher risk of population recovery^17,19,28^.

The advent of thermal-imaging technologies, and their increasing availability and quality, provide an opportunity to improve the detection of animals at low densities or in low-detectability habitats. Thermal-imaging equipment has been used in wildlife monitoring surveys since the 1960s [see^29,30^], mostly for the detection of large ungulates, but also recently for surveys of macropods^31^, koalas (*Phascolarctus cinereus*)^32^, and spider monkeys (*Ateles* spp.)^33^, as well as in the detection of active rabbit warrens^34^. When used correctly, thermal-imaging equipment can detect more animals than do visual surveys^31,32^, and detects fossorial animals and their burrows more efficiently even when obscured by vegetation^34,35^.

Thermal imaging technologies have been used successfully in ground-shooting campaigns to control many species including rhesus macaques (*Macaca mulatta*)^36^, sambar deer^37^, and kangaroos (Macropodidae)^38^. However, there is little published information on the use of thermal equipment in aerial culling programs. Recently, thermal imaging paired with shotguns was successfully trialled on fallow deer in south-east South Australia^5^. There is one published record of using thermal imaging in aerial culling for pig eradication at Hawaii Volcanoes National Park^39^, although there is no detail on how it was used. Thermal imaging has also been trialled in several formats for aerial culling in New Zealand since 1982, and extensive research and development on its use in aerial culling has occurred since 2015 [N. McDonald, unpublished data, 2018]. The developed method of thermal aerial culling (also known as thermal-assisted aerial culling and thermal-assisted aerial shooting), has been used to remove pigs from Falla Peninsula on Auckland Island [F. Cox, unpublished data, 2019], red deer from Five Fingers Peninsula on Resolution Island [N. McDonald, unpublished data, 2018], and goats from Raukumara Ranges (D. Paine, personal observation, 28 May 2022).

Thermal technology has the potential to improve the detection of animals in many habitat types. More pigs and deer (*n* = 533) were found by a dedicated thermal operator than by visual observers (*n* = 302) during an aerial cull on the Hay Plains in New South Wales across habitat types (T. Cox, unpublished data, 2021) Additionally, using thermal technologies in the thermal aerial culling configuration could enable following multiple animals during a pursuit (by the thermal-imager operator while the shooter is focused on the target), thereby reducing the likelihood of losing other animals in the group. More efficient tracking could also improve animal welfare by reducing the probability that animals are shot/wounded and then escape to thick vegetation before incapacitation is confirmed^5^.

The death of an animal can be difficult to confirm from the air. We instead use incapacitation (animal is recumbent, immobile and regarded as unconscious) to indicate ‘apparent death’, recognizing that an incapacitated animal may not be clinically dead^40^. Hampton et al.^41^ described four parameters that could be quantified to assess welfare outcomes for helicopter- and ground-based shooting programs^42^(modified for our use of incapacitation); (1) wounding rate (proportion of animals shot but not incapacitated), (2) time to incapacitation, (3) instantaneous incapacitation rate, and (4) anatomical locations of bullet wounds.

We present data on two thermal aerial culling operations in Australia. We describe the efficacy of the method for two vertebrate pest species: a low-density (< 0.2 animals km^−2^) population of feral pigs, and a high-density (> 6 animals km^−2^) population of feral fallow deer. The pig population had been reduced by 90% during the 2019–2020 bushfires^17,43^ and this presented a unique opportunity to attempt to eradicate the low-density population while vegetation recovery enabled high detectability. We measured several aspects of the approach to test the following hypotheses: (*i*) the number of shots taken and shots impacting an animal do not vary relative to vegetation-density category (open, sparse, dense) or species (pig, deer), (*ii*) four temporal welfare indicators (time from start of pursuit to first bullet impact [chase time], time from first bullet impact to incapacitation, time from first shot to incapacitation, time from pursuit to incapacitation) do not vary among vegetation-density categories and species, and (*iii*) no wounded animals escaped. We also discuss the impact of crew configuration on target acquisition and tracking and the use of thermal technology to mitigate negative animal welfare outcomes. Because we did not inspect carcasses, we could only collect information on wound rate and time to incapacitation.

## Materials and methods

### Location and target species

**Figure 1:**
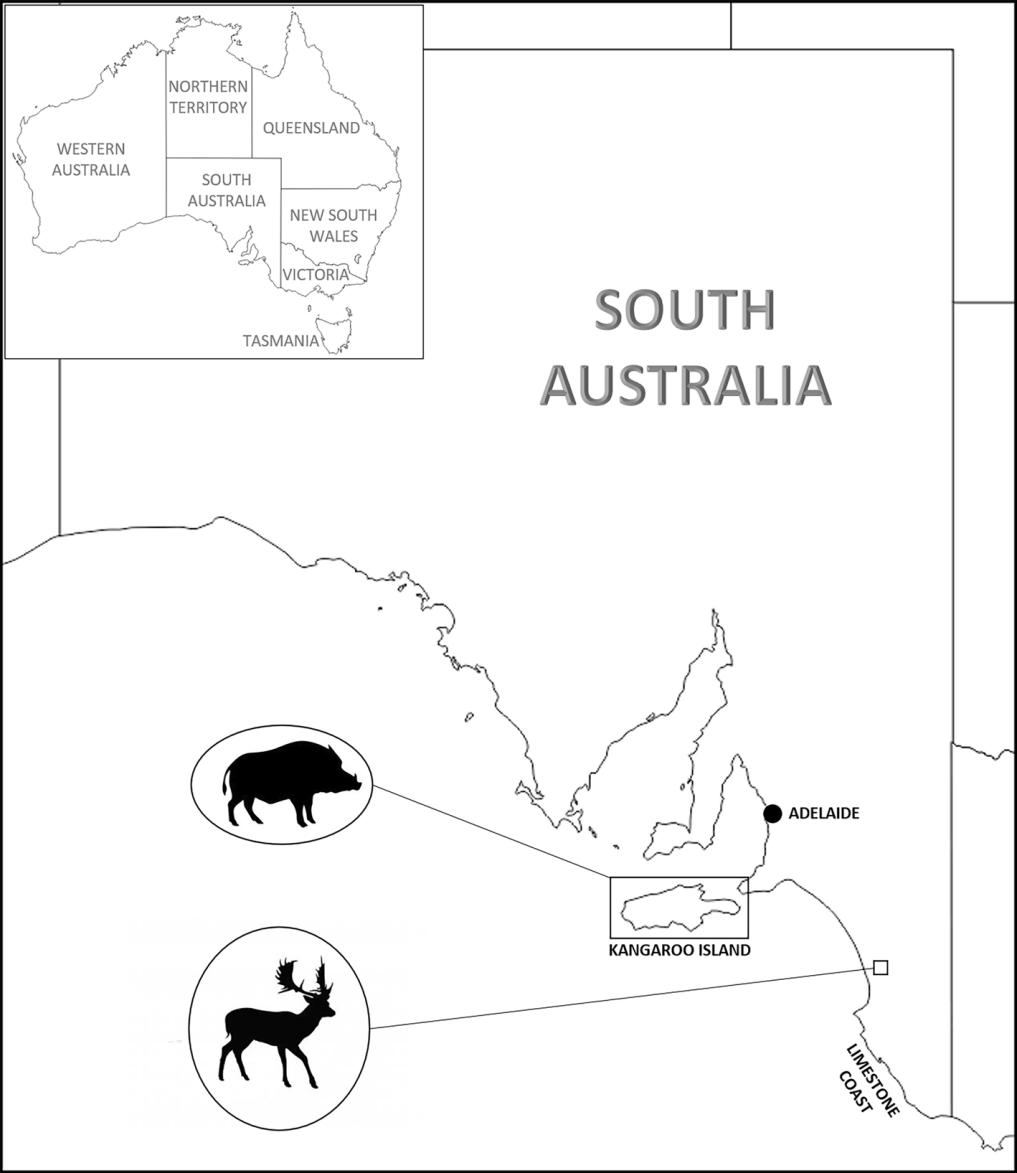
Location of the two thermal-assisted aerial culling programs done in South Australia: pigs on Kangaroo Island, and fallow deer near Kingston in the Limestone Coast region.

We did two thermal aerial culling programs in South Australia (Fig. 1) during 2021: pigs on Kangaroo Island (18–30 March) and fallow deer on the Limestone Coast in south-eastern South Australia (27–29 September). The animals were never handled but they were shot and killed from the air. They were culled as part of an ongoing pest management program from which we collected data**.**

Kangaroo Island (*Karta Pintingga*) is Australia’s third-largest island, 112 km south of Adelaide, South Australia (Fig. 1). The island has a terrestrial area of 4405 km^2^ and is 145 km long (east–west) × 54 km wide at its widest point, with a population of > 4000 people. Over one third of the island is protected in nature reserves. Decades of intense feral animal control has eradicated both deer and goats^23^. Eradication is now focused on feral pigs^17,43^ and cats (*Felis catus*)^44^. After the 2019–2020 fires, the pig population was estimated at < 450 individuals^43^. As part of the Island’s post-bushfire pig-eradication program, there had been extensive ground shooting (one intensive, three-month period that removed 165 pigs), trapping, and baiting for pigs across the island. Ad hoc visual aerial culling occurred in March 2020 while a helicopter was on the Island for aerial baiting. This approach resulted in three 2-h shooting sessions and removed 7 pigs (1.2 pigs hour^−1^). The remaining individuals were increasingly difficult to bait or trap, with animals restricted to the western end of the Island, and typically located in inaccessible parts of parks and reserves, former plantation forests, as well as farms. The thermal program consisted of 36.0 flying hours over the western part of the Island in previously identified areas of high pig activity^43^.

The Limestone Coast is a low-lying sand dune region of south-eastern South Australia. Feral deer occur throughout the Limestone Coast region with fallow deer the most common. The thermal aerial culling program had a total of 15.5 flying hours across bushland on seven private holdings covering 173.7 km^2^. Properties included a mix of grazing and cropping, with areas of remnant tea tree (*Melaleuca alternifolia*) and eucalypt woodland. Visual shoots have been done in this area 1–2 times year^−1^ for several years. Landholders also engaged contract, volunteer, and/or recreational shooters to assist with deer management and a commercial harvester also worked across most of the seven holdings regularly between 2018 and 2020. A visual aerial cull was done over the area 10 days prior (13–17 September) to the thermal aerial culling program. The visual shoot crew flew 89.9 h, using 2 helicopters over 5 days, and culled 603 deer (6.8 deer hour^−1^) across 128,103 ha, with the aim of removing as many deer as quickly as possible. While vegetated areas were included, targeting occurred mainly on open pasture.

### Thermal aerial operations

Thermal aerial operations were generally done in the 2 h from first light and 1.5 h before last light each day, weather permitting, when there was the greatest difference in thermal radiation between animals and their surrounding environments (ΔT). When weather was suitable (cool and overcast with high cloud) flights could continue throughout the day. Low-level night-time operations such as shooting are not permitted by the Australian or New Zealand civil aviation authorities.

**Figure 2:**
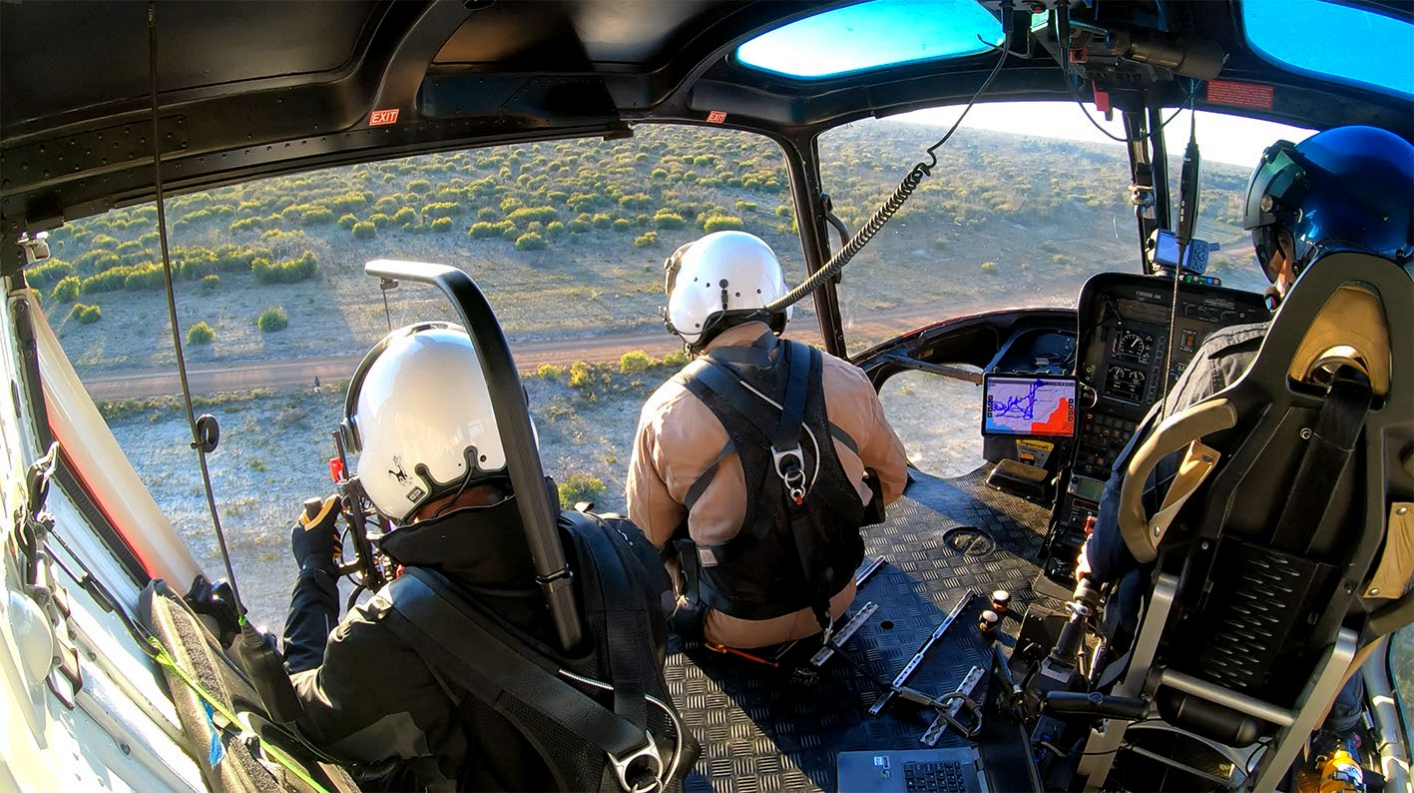
Crew configuration of thermal aerial culling in an AS350 B3 Squirrel helicopter. The pilot is in the front right, shooter in the front left, and thermal operator in the rear left. The shooter and the thermal operator are secured to the helicopter using approved aviation quick-release harnesses.

Thermal aerial culling comprises a specific crew configuration (a pilot, a shooter who sits opposite the pilot, and a thermal-imager operator who sits behind the shooter) (Fig. 2). All other uses of thermal equipment (e.g., scopes for rifles, or thermal monocular/binoculars) in aircraft in any other crew configuration, we consider a hybrid aerial culling approach.

During a flight, both the shooter and thermal operator sat on the floor and were tethered to the helicopter using approved aviation quick-release harnesses (Fig. 2). The tether was as short as possible, yet long enough to allow the crew member to lean over to see the ground beneath the helicopter. We set the thermal imager to output ‘white-hot’ greyscale imagery, which was the operators preferred output.

**Figure 3:**
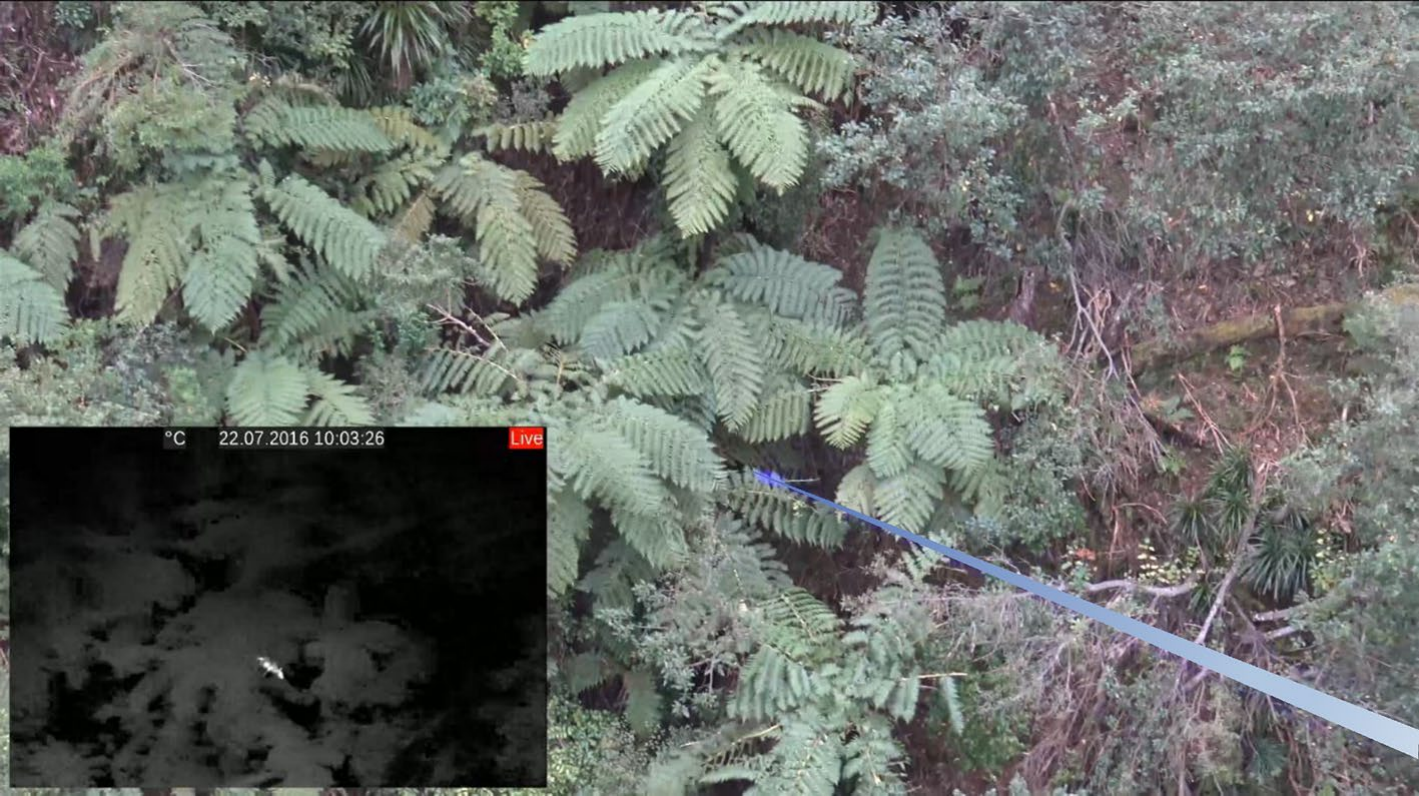
A target animal hiding under dense vegetation seen by the thermal operator (inset) and located for the shooter with a laser (blue).

The aircraft (AS350 B3 Écureuil [Squirrel] helicopter; Aérospatiale, France) was positioned at approximately 50–100 m above ground level and flew at 15–25 knots ground speed. The helicopter searched the area using a pseudo-systematic search pattern: the area was flown in parallel transects allowing for thorough coverage and the helicopter deviated from the search pattern to pursue target animals. When a potential target was detected, the thermal operator verbally alerted the shooter and pilot, providing information on the suspected species and its general direction/location. The thermal operator also identified the target’s location to the shooter and pilot by switching on a 12 V, 1 W, 520 nm focusable laser pointer (Oxlasers, Shanghai, China) (Fig. 3). The shooter carried handheld thermal binoculars and used these to confirm the location and species of the detection. Where detections were obscured by vegetation (i.e., something warm was detected but identification could not be determined), the pilot would reposition the helicopter, generating rotor wash to flush the animal so it could be identified. During these programs the shooter used a Springfield M1A 0.308—calibre self-loading rifle with 135 grain hollow-point ammunition for pigs, and 150-grain soft-point ammunition for deer. Incapacitation of animals had to be confirmed by both the shooter and the thermal operator and was based on cessation of movement and wound placement.

### Imaging equipment

In both Australia and New Zealand, the Sierra-Olympic Vayu HD uncooled microbolometer array has been used in these types of programs. This imager has a 24 × 14.5 mm sensor that produces a 1920 × 1200-pixel image and has a refresh rate of either > 30 (Generation 1, New Zealand) or 60 Hz (Generation 2, Australia). We viewed output from the thermal imager on a Blackmagic Video Assist 7″ 12G HDR monitor (blackmagicdesign.com/au/products/blackmagicvideoassist) and recorded video onto SD cards for later review. A Panasonic GH5 4 K video camera was also mounted to the top of the thermal imager to collect comparative visual footage of animal detections. The imager and monitor were mounted to a custom-built frame that was then suspended using an Easyrig Minimax camera mount (easyrig.se) worn by the thermal operator. The laser pointer was mounted to the frame of the thermal imager and calibrated to point to the centre of the field of view. The thermal operator used the laser to identify visually the location of detected animals to the shooter (Fig. 3). The shooter was also equipped with a thermal scope (either clip on or interchangeable with the visual scope) and carried handheld thermal binoculars or a monocular to assist with searching and species identification.

### Data and analysis

We recorded each flight with the Blackmagic Video Assist (thermal), a Panasonic GH5 4 K video camera and a GoPro 3 camera mounted to the rear firewall of the helicopter. Both the Blackmagic and the GoPro recorded the flight audio. Audio was used to determine the total number of shots taken at an animal, and the time between first shot and last shot. Audio was used in conjunction with the thermal video footage to determine which and how many shot/s impacted the animal. We used the GoPro audio when the audio on the Blackmagic recording was unclear or did not record. Where no audio recording was usable, no information on the number of shots or the time from first to last shot could be collected. Experienced personnel (with > 50 h experience logging thermal-imaging surveys) reviewed and logged the thermal footage. Two experienced personnel reviewed footage for both pigs and deer to reduce any bias and to ensure accuracy.

We transcribed all footage in Excel® including (1) group size, (2) time animal(s) were first detected, (3) time pursued, (4) habitat type where detected (dense = tall timber or shrub canopy with no ground visible, sparse = tree or shrub canopy with ground visible, open = no tree or shrub canopy), (5) time of first-last shots taken, and (6) number of impact shots until incapacitation was confirmed. Where the footage did not show the impact shots, we excluded these data from analysis. We only considered ‘detected’ animals that could be seen on the thermal footage. Some animals were seen visually; however, we could not verify the number of animals because there was no associated recording of those observations.

We used data collected from the thermal imagery to measure five aspects of the thermal aerial culling approach to infer the duration and intensity of suffering of animals: (1) time between first and last shot, (2) time from first impact shot to incapacitation, (3) time from start of the pursuit until first impact shot (chase time), (4) time from start of the pursuit until last shot, and (5) total number of impact shots. We used the Analysis Toolpak in Excel® to generate summary statistics. While we provide median values as a more accurate representation of duration of events, we also present means for some data to allow comparison to other published studies.

We constructed a series of generalised linear models using the *glm* function in the stats R library^45^ to test for the relative effects of habitat type (open, sparse, dense [vegetation]) and species (pig or deer) on the shots (number of shots taken; number of impact shots). We applied a Poisson error distribution and a log link-function, and log_10-_transformed the time data with a Gaussian error distribution. For each variable we contrasted five models, including the two additive main effects, their interaction, single effects, and the intercept-only model. We compared the relative probability of the five models per response variable using Akaike’s information criterion corrected for small sample size (AIC_c_)^46^. The bias-corrected relative weight of evidence for each model, given the data and the suite of candidate models considered, was the AIC_c_ weight (the smaller the weight, the lower its contribution to parameter estimates)^46^. We also calculated the percent deviance explained (%DE) as a measure of goodness of fit. For the habitat models, there was a single entry in the ‘open’ category for pigs, so we removed that category from all analyses. We also constructed Kaplan–Meier survival curves using the survival package^47^ in R.

## Results

**Figure 4:**
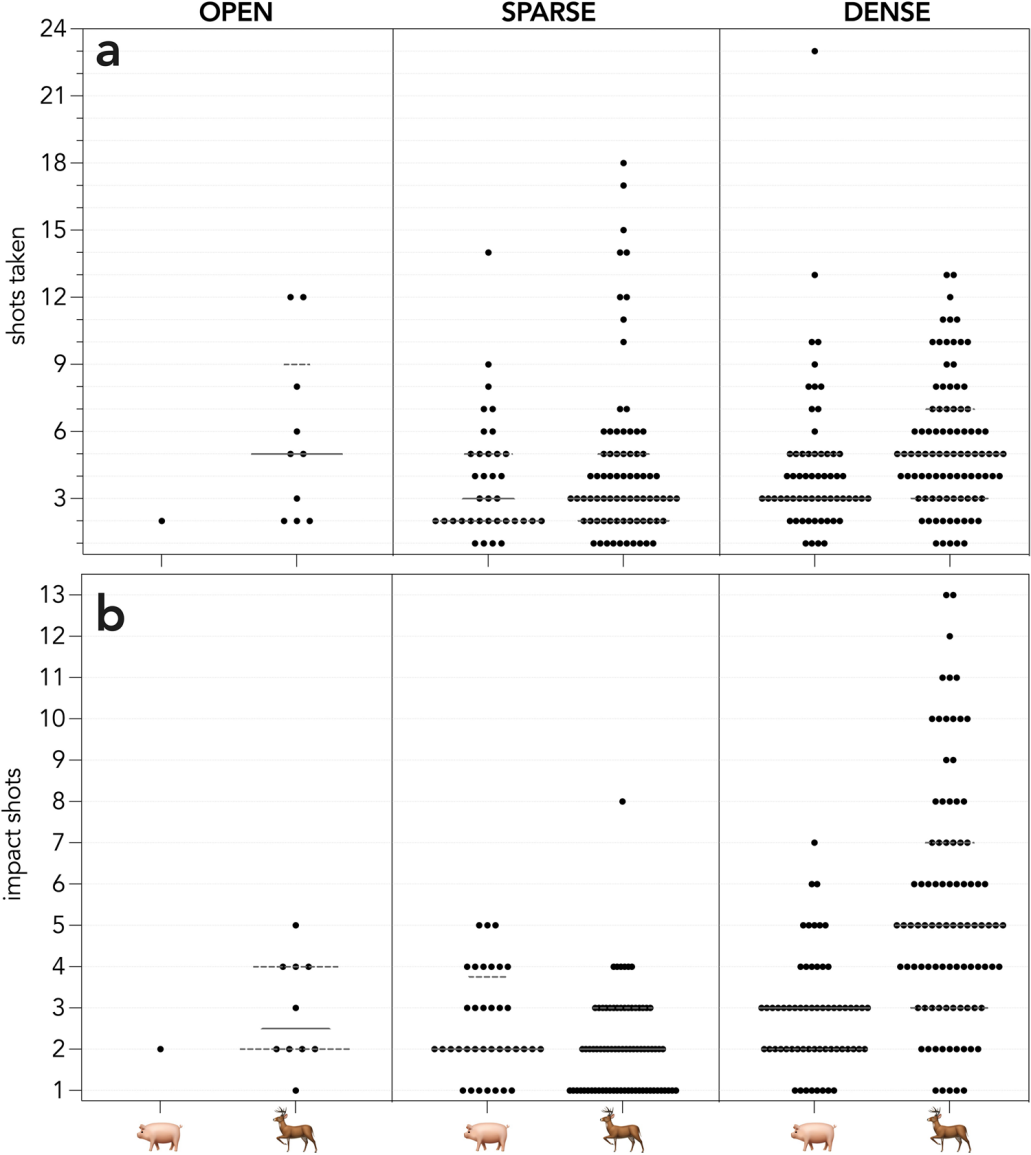
Number of shots (black dots) that (**a**) were fired at an animal and (**b**) that impacted the animal across the three habitat types (OPEN, SPARSE vegetation, DENSE vegetation) in which animals were first seen.

**Figure 5:**
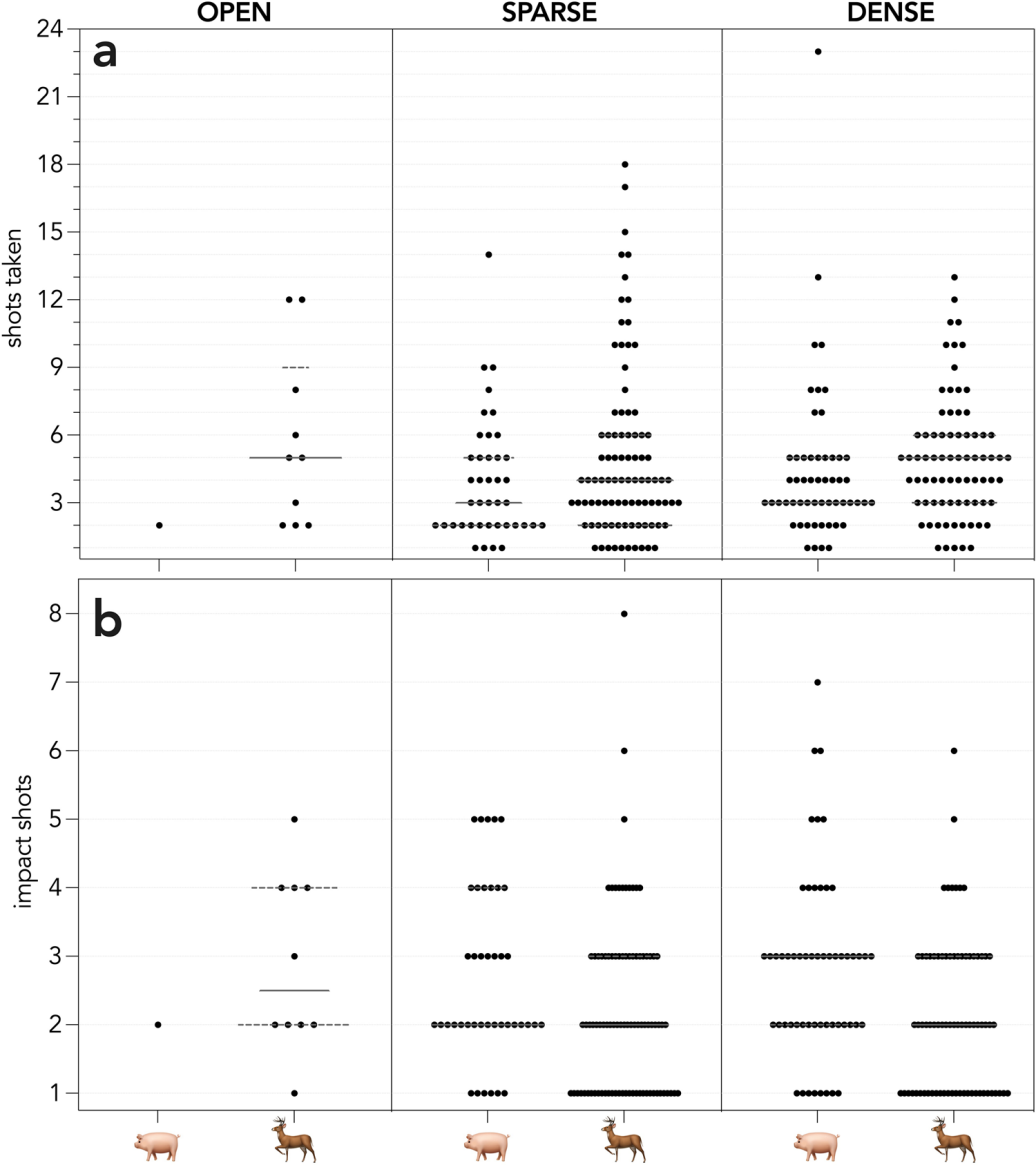
Number of shots (black dots) that (**a**) were fired at an animal and (**b**) that impacted the animal across the three habitat types (OPEN, SPARSE vegetation, DENSE vegetation) in which animals were shot.

On kangaroo Island, 138 pigs were detected, and 122 were culled; a cull rate of 3.4 pigs hour^−1^. All except six pigs were culled in the habitat type in which they were detected, with those six pigs moving from a dense to sparse habitat. Only one pig was detected (and shot) in an open habitat. On the Limestone Coast, 246 deer were detected, and 188 were culled resulting in a cull rate of 12.1 deer hour^−1^. All except 15 deer were culled in the habitat type in which they were detected, with those 15 moving from a dense to sparse habitat (cf. Figs.  4, 5). Sixteen pigs and 55 deer escaped, of which 13 (pigs) and 41 (deer) were seen and escaped, 1 (pig) and 14 (deer) were not pursued due to the pursuit of other animals or poor light conditions (pigs = 2). Three deer moved beyond the shoot boundary and their pursuit was abandoned. Mean group size was similar for both species (pigs = 2, largest group size = 6; deer = 2, largest group size = 8), and many single individuals of both species were pursued. Seventy-two percent (pigs) and 53% (deer) were first detected in dense habitats.

There were insufficient data (audio and thermal video) to determine accurate and detailed shooting data for 24 pigs and 8 deer. For the remaining 98 pigs and 180 deer, animals were shot at a median of 3 (SD = 3) and 5 (SD = 3.7) times with median impact shots of 2.5 (SD = 1.3, mean = 2.7) and 2 (SD = 1.2, mean = 2.2), respectively (Fig. 4).Fifteen pigs and 13 deer were confirmed incapacitated after a single impact shot. Median chase time was 71 s (SD = 171.2, mean = 110.8) for pigs and 47.0 s (SD = 56.7, mean = 62.5) for deer. Median time to incapacitation from the first impact shot was 11.5 s (SD = 42.7, mean = 24.2) for pigs and 11.0 s (SD = 33.2, mean = 21.9) for deer, with a median pursuit time until incapacitation of 94.5 s (SD = 182.6, mean = 145.5) and 98 s (SD = 71.4, mean = 107.7) respectively. Each animal impacted by a bullet was confirmed incapacitated across both programs. For both species, the wound rate = 0, resulting in a incapacitation efficiency of 100%.

There was only weak evidence (% deviance explained ≤ 7.5%) for a difference in the total number of impacting shots among habitat types in which animals were first seen (Table 1). There were approximately equal contributions of habitat type and species, and a weak effect of altitude above ground at time of impact (Table 1).

**Table 1:** Top-ranked subset (including single-term models) of the generalised linear models to test for the effects of habitat in which first seen(H) type (open, sparse, dense [vegetation]), species (S) (pigs or deer), and altitude above ground at time of impact (A) on the number of shots impacting the animal. * denotes an interaction term. *k* = number of model parameters; $\ell$ = −log likelihood; AIC_*c*_ = Akaike’s information criterion corrected for small sample size; *w*AIC_*c*_ ≈ model probability; %DE = percent deviance explained.

Model	*K*	$\ell$	AIC_*c*_	*w*AIC_*c*_	%DE
~ H + S	4	− 450.061	908.268	0.367	7.2
~ H + S + A	5	− 449.335	908.890	0.268	8.0
~ H + S + A + S*A	6	− 449.146	910.601	0.114	8.2
~ H + S + A + H*A	7	− 448.918	912.251	0.050	8.4
~ H + S + A + H*S	7	− 448.957	912.329	0.048	8.4
~ H	3	− 453.221	912.530	0.044	3.6
~ S	2	− 456.847	917.738	0.003	3.6
~ A	2	− 458.563	921.171	< 0.001	1.7

**Figure 6:**
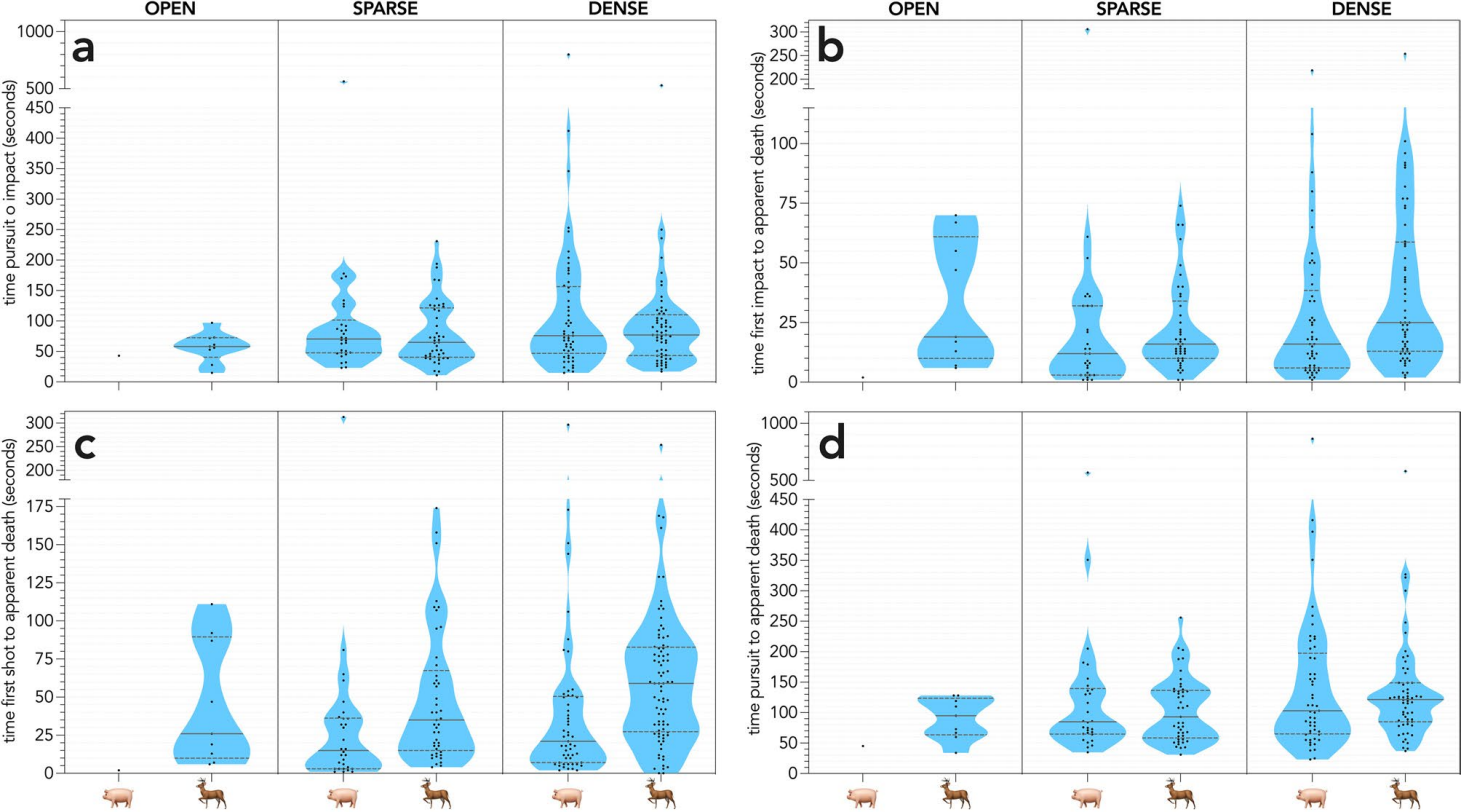
Interval times (in seconds) across three habitat types (OPEN, SPARSE vegetation, DENSE vegetation). (**a**) time from start of pursuit to first bullet impact; (**b**) time from first bullet impact to incapacitation; (**c**) time from first shot to incapacitation; (**d**) time from pursuit to incapacitation.

The evidence for an effect of habitat type in which first seen and species on the time intervals (Fig. 6) varied depending on the interval used as the response variable. There was no evidence for any effect on the time between pursuit and impact, but a moderate effect of both habitat and species on the time between being shot and incapacitation (driven mainly by differences between the two species) (Table 2). The two remaining response variables (time between impact and incapacitation; time between pursuit and incapacitation) had comparatively weak effects of both variables, with approximately equal contributions of both for the former, and habitat type for the latter (Table 2).

**Table 2:** Top-ranked subset of the generalised linear models to test for the effects of habitat where first seen (H) type (open, sparse, dense [vegetation]) and species (S) (pigs or deer) on the time intervals between pursuit and impact, impact and incapacitation, shot and incapacitation, and pursuit and incapacitation. * denotes an interaction term. *k* = number of model parameters;  = −log likelihood; AIC_*c*_ = Akaike’s information criterion corrected for small sample size; *w*AIC_*c*_ = model probability; %DE = percent deviance explained.

Model	*k*	$\ell$	AIC_*c*_	*w*AIC_*c*_	%DE
Pursuit–impact					
~ 1	1	− 49.585	102.231	0.279	–
~ H	3	− 47.535	103.278	0.273	2.1
Impact–apparent death					
~ H + S	4	− 134.723	279.759	0.583	7.9
~ H + S + H*S	6	− 133.250	281.090	0.300	9.3
Shot–apparent death					
~ H + S	4	− 132.035	274.382	0.692	17.1
~ H + S + H*S	6	− 130.848	276.285	0.267	18.1
~ S	2	− 136.966	280.056	0.041	12.9
Pursuit–apparent death					
~ H	3	− 11.978	32.163	0.550	3.3
~ H + S	4	− 11.965	34.242	0.194	3.3

**Figure 7:**
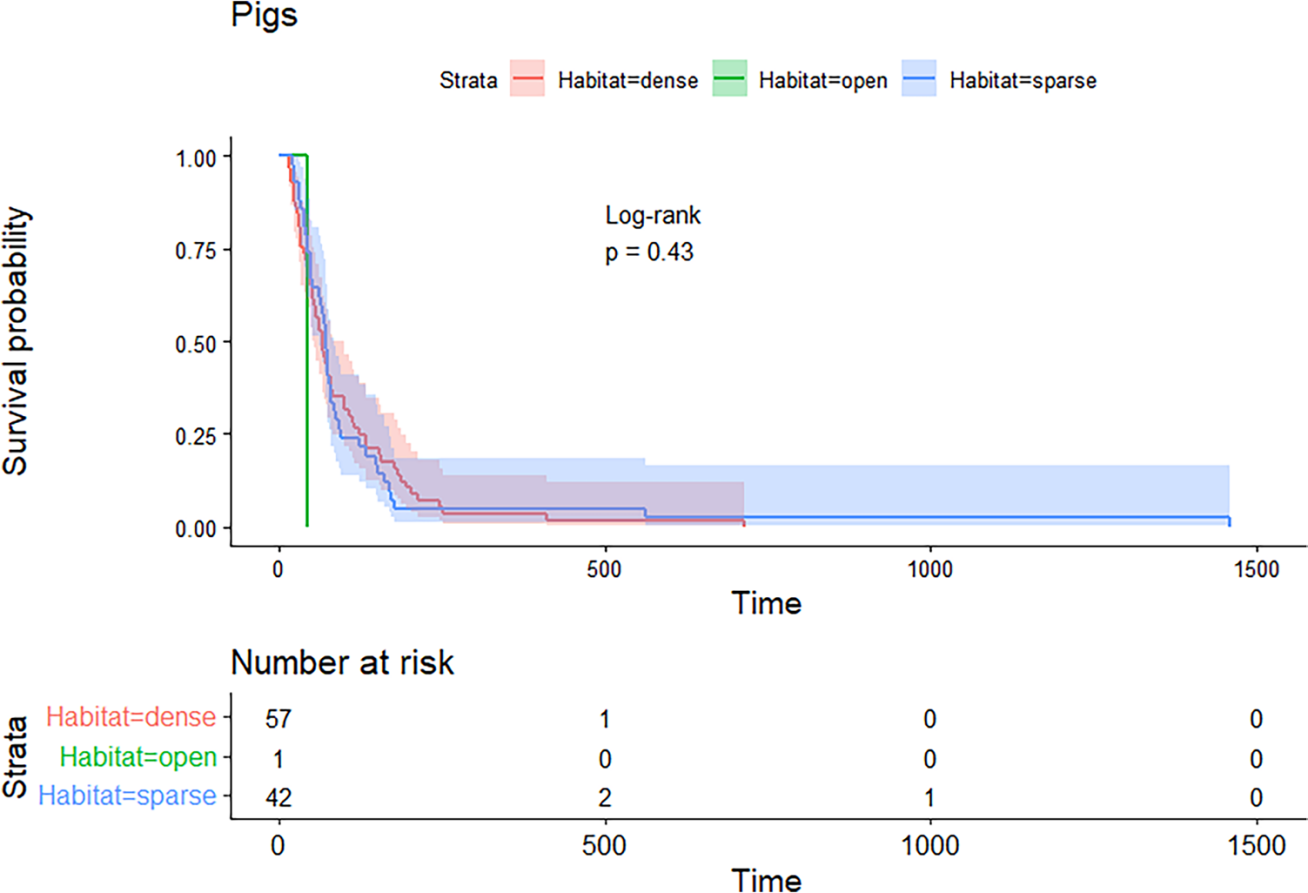
Survival probabilities and times (s) and the effect of habitat on pig survival during a shooting program using the thermal aerial culling crew configuration (*χ*^2^ = 1.7, *df* = 2, *p* = 0.4). Shading represents 95% confidence intervals.

**Figure 8:**
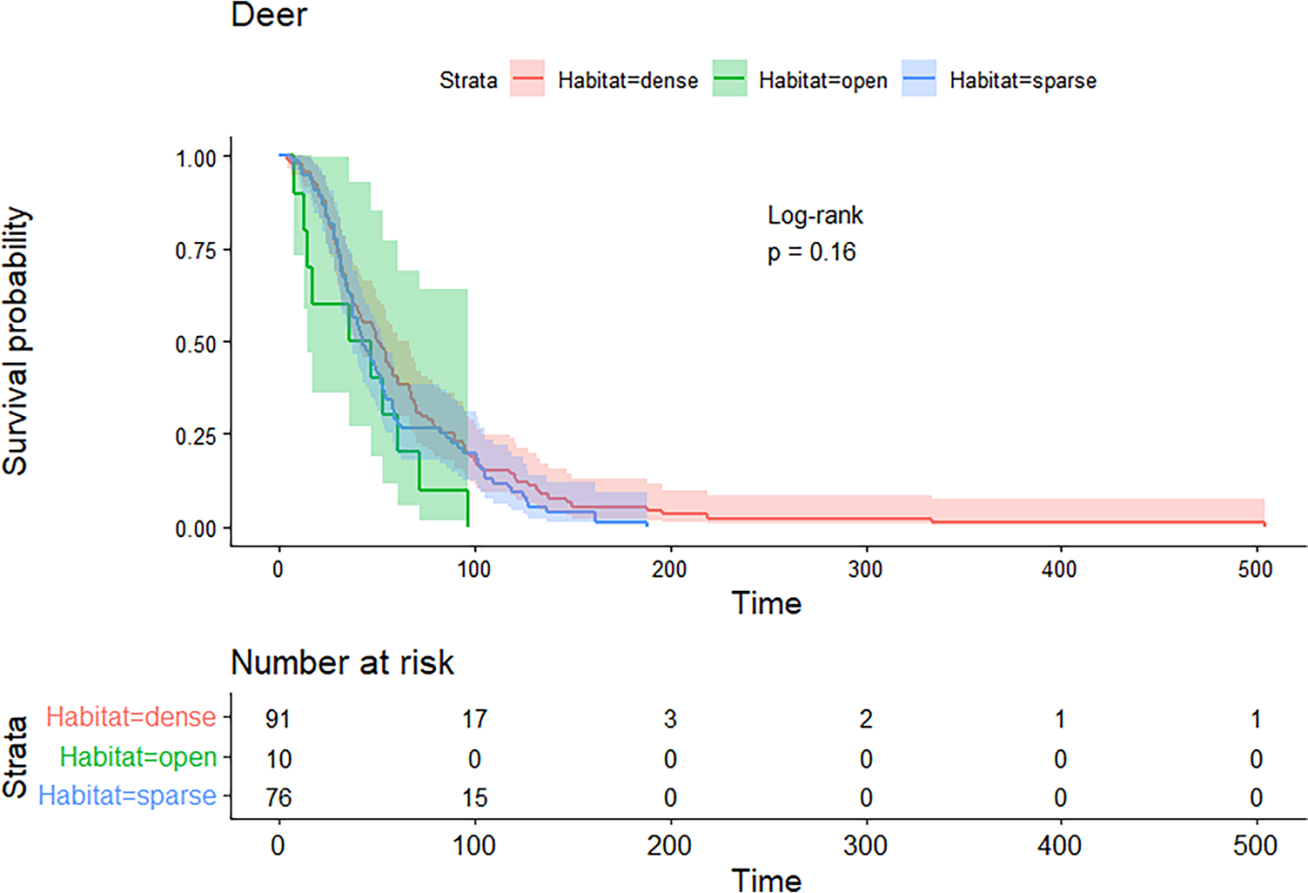
Survival probabilities and times (s) and the effect of habitat on deer survival during a shooting program using the thermal aerial culling crew configuration (*χ*^2^ = 3.7, *df* = 2, *p* = 0.2). Shading represents 95% confidence intervals.

For the Kaplan–Meier survival analysis, habitat data were missing for three deer. Survival times are shown for each species in each habitat type, but habitat type had no effect on survival for either pigs (*χ*^2^ = 1.7, *df* = 2, *p* = 0.4) (Fig. 7) or deer (*χ*^2^ = 3.7, *df* = 2, *p* = 0.2) (Fig. 8).

## Discussion

Our results confirm that thermal imaging can be used to detect pest animals during aerial culling in difficult habitat and that the use of thermal equipment in the thermal aerial culling configuration can increase the culling rate of an aerial shooting program. For both species the culling rate was doubled in the thermal aerial culling configuration (pigs = 3.4 h^−1^, deer = 12.1 h^−1^) compared to the rate during visual shoots over the same populations (pigs = 1.2 h^−1^, deer = 6.8 h^−1^). Most importantly, our data show that the wound rate was zero, resulting in a 100% incapacitation efficiency. Wounded and escaped individuals have been reported during visual aerial culling programs for deer^48^.

There was no discernible effect of habitat type on the detection and subsequent dispatch of target animals for either species. However, there was a moderate effect of both habitat type and species type on time to incapacitation. These effects can likely be attributed to species behaviour. Both populations had been subject to previous aerial control and previous studies report that experienced animals will evade aircraft by seeking shelter [e.g.,^16,19,49–51^, but see^52^]. This behaviour and the observation most of the detected animals were in dense habitat, probably increased time to incapacitation. However, the metrics we report for deer are comparable to those reported during a welfare assessment of visual aerial culling programs for deer^48^; we report a similar number of average impact shots (2.2, SD = 1.2) compared to 1.43–2.57 for the visual-only^48^, potentially shorter chase times (62.5 s, SD = 56.7 vs. 73–145 s, respectively), similar average total times (107.7 s, SD = 71.4 vs. 109–162 s, respectively) and comparable times from first shot to last shot (21.9 s, SD = 33.2 vs. 17–37 s, respectively). Welfare assessments for visual helicopter-based aerial culling have not been done for pigs, but the number of impact shots pig^−1^ in a visual aerial culling study assessing ammunition were similar (2.7, SD = 1.3 vs. 2.98–3.29)^53^.

Our data suggest that using thermal imaging in this configuration to track animals improves animal welfare outcomes. Positioning the thermal operator and the shooter on the same side maximised target detection and dispatch and prevented wounded animals from escaping. The number of animals seen and escaped was reduced compared to previous studies—only 9% of detected pigs and 16% of deer were seen and escaped, considerably fewer than for visually targeted deer where 24–31% were seen and escaped^48^. Additionally, the reported number of animals seen and escaped during our study is potentially overestimated. When density is high, such as on the Limestone Coast, it is difficult to track all individuals in a group. During a shoot, individual deer would often leave the group or additional deer would be acquired. Without being able to identify animals individually, it is difficult to know if escaped animals are in fact seen and escaped, or whether they are reacquired as part of another group. For this reason, ‘seen and escaped’ data from the deer shoot are probably inaccurate. Similarly, for pigs, the number of seen and escaped animals is likely an overestimation. While some individuals were seen and escaped, often revisiting that area the next day would result in the removal of the same number of animals that were seen and escaped in the area the day before. It is likely that these were the same individuals.

The crew configuration used in this study can be particularly useful when detection probability is low due to dense vegetation, avoidance behaviour, or low density. Having two sets of thermal (operator and shooter) scanning the environment increases the chances of detection, reduces the chances of evasion and increases the efficiency of the program. It also assists in searching for shot animals to ensure incapacitation, particularly in dense vegetation where visual detection is not possible. Using thermal imaging equipment in this configuration could provide high confidence in aerial culling programs, especially those that are focused on eradication.

## Conclusion

The use of thermal-imaging technology in the thermal aerial culling configuration increases the detection of animals in aerial culling programs and can substantially increase the culling rate of such programs when compared to visual aerial culling. The crew configuration we used maximised target acquisition and re-acquisition opportunity, reduced the percentage of animals seen and lost, and resulted in no loss of wounded animals, thereby improving the animal welfare outcomes normally associated with aerial culling. The ability to detect animals in difficult habitat, that are at low densities, or that exhibit avoidance behaviour, coupled with the ability of the thermal operator to track and monitor additional members of a group will improve the success of control and eradication programs and ensure the best possible animal welfare outcomes.

## Data Availability

The data used in the analysis is available from the corresponding author. The original thermal footage is the property of The Department of Primary Industries and Regions South Australia.
